# The Structure of Demand, Control, and Stability-Support Underlying the Job Content Questionnaire (JCQ) 2.0—An Innovative Tool for Assessing Multilevel Work Characteristics

**DOI:** 10.3390/ijerph22091403

**Published:** 2025-09-08

**Authors:** Maren Formazin, BongKyoo Choi, Maureen F. Dollard, Jian Li, Sarven S. McLinton, Wilfred Agbenyikey, Sung-il Cho, Irene Houtman, Robert Karasek

**Affiliations:** 1 Federal Institute for Occupational Safety and Health (BAuA), Division “Work and Health”, 10317 Berlin, Germany; 2 Center for Work and Health Research, Irvine, CA 92620, USA; 3 Department of Medicine, University of California, Irvine, CA 92617, USA; 4 PSC Global Observatory, University of South Australia, Adelaide 5000, Australia; 5 Department of Environmental Health Sciences, Fielding School of Public Health, School of Nursing, University of California, Los Angeles, CA 90095, USA; 6 US Department of Health and Human Services, Center for Medicare and Medicaid Services, Baltimore, MD 21244, USA; 7 Kintampo Campus, Martin Luther Health Training School, Kintampo P.O. Box 176, Ghana; 8 Department of Public Health Science, School of Public Health, Institute of Health and Environment, Seoul National University, Seoul 08826, Republic of Korea; 9 TNO Netherlands Organization for Applied Scientific Research, 2333 BE Leiden, The Netherlands; 10 Institute for Psychology, Copenhagen University, 1353 Copenhagen, Denmark; 11 Department of Work Environment, University of Massachusetts Lowell, Lowell, MA 01854, USA; 12 Øresund Synergy, 1820 Copenhagen, Denmark

**Keywords:** demand, control, stability-support, structure, structural equation modeling, JCQ 2.0, DCS model

## Abstract

Dominant theories in the field of occupational stress have so far mainly focused only on job task level psychosocial factors. Our novelty was to move the field forward by testing a new multilevel conceptualization of workplace health-related psychosocial factors, captured in the new JCQ 2.0 tool. The JCQ 2.0 tool assesses the theoretical constructs Demand (D), Control (C) and Stability-Support (S-S) at the task and the organizational level in accordance with the Associationalist Demand/Control (ADC) Model. We aimed for a first step to assess the generalizability of the framework by collecting data in four different countries (Korea, China, Australia, and Germany). Using structural equation modeling, the task level three-factor DCS structure was largely confirmed across all four countries (with one exception: skill discretion was an indicator of both control and demand). The organizational level, three-factor DCS-S structure was tested and confirmed in the German data only (only data with sufficient scales). Similarly, the multilevel DCS-S model could only be tested with the German data only and was largely confirmed with the three organizational level factors (D, C, and S-S) as antecedents to their task level analogues (with one exception: supervisor support was an indicator of organizational rather than task level support). The findings provide a first step to advancing existing knowledge by providing preliminary support for a multilevel DCS model. Further multilevel longitudinal research is required to verify the main findings and explain some of the nuances uncovered here.

## 1. Introduction

In the past, influential theories in the field of occupational stress have focused largely on task level psychosocial factors. The demand control model (DC-model) as originally proposed by Karasek [1] is a well-established work stress model which describes two underlying task-related concepts: demand and control. In the 1980s, this model was expanded with a third dimension: social support (S) [2,3]. In order to assess these three task level dimensions, the Job Content Questionnaire (JCQ) [4] was developed with its latest version JCQ 1.7 dating from 1996. The Job Content Questionnaire JCQ 1 has been widely used in research on work stress all over the world with versions available in more than 25 languages [https://www.jcqcenter.com/questionnaires-jcq-jcq2/ (accessed on 1 April 2025)]. The utility of the DCS model has been significant for predicting important work outcomes, demonstrated in several meta-analyses [5,6], especially with regard to lagged causal effects of work characteristics on (self-reported) health and wellbeing [7] and for informing humane work redesign.

However, changes in the world of work have been tremendous in recent times due to digitalization, the growth of the service sector, and globalization. Effects of these developments influence people all over the world. For example, the Suez Canal blockage due to a ship grounding accident in 2021 had a severe impact on delivery chains around the globe, affecting the economy and, hence, workers on all continents and triggering research on the global shipping network [8]. Similarly, current considerations regarding the introduction of tariffs create uncertainty for companies [https://www.europarl.europa.eu/topics/en/article/20250210STO26801/eu-us-trade-how-tariffs-could-impact-europe (accessed on 18 July 2025)], and consequently, rising job insecurity as well as a worsening of the working conditions for workers. Moreover, in interviews with workers reporting high burnout ratings—conducted by one of the co-authors of this paper—one respondent, an IT engineer, said that he dropped out with work-related mental health problems due to increased work pace and lengthened working time (>11 h/day). The latter were a result of the high competition his employer—a German automobile factory—faced from Chinese factories.

In line with these observations, mental health at work has gained more and more attention in recent years and decades: the World Health Organization has clearly stated “Decent work is good for mental health” [https://www.who.int/news-room/fact-sheets/detail/mental-health-at-work (accessed on 18 July 2025)] and has published the “WHO guidelines on mental health at work” [9] with recommendations to promote mental health, prevent mental health conditions, and enable people living with mental health conditions to participate and thrive in work. These guidelines have been complemented with the “Mental health at work: policy brief” [10] meant for governments, employers, employers’ and workers’ organizations, civil society and health service planners to guide action on mental health at work.

Considering these developments, one has to state that the JCQ 1, based on the DCS model and originating from the 1980s, does not assess all working conditions relevant for workers’ mental health in today’s world of work. For example, it does not contain scales assessing emotional demands—especially present in the service sector which has grown steadily over the last decades—or negative acts like bullying. Further, there is a need to move beyond task level factors to recognize organizational influences in workplaces.

Since the early 2000s, the DCS model has been developed further by Karasek and colleagues and has been expanded to the Associationalist Demand/Control Model (ADC). The latter not only considers the task level—as the DCS model has done before—but extends this further to take account of the organizational level as well as the external-to-work level. Additionally, it expands social support to stability-support (S-S). Karasek et al. [in this Special Issue; see Appendix A, A.1] describe both the development process and ADC theory in detail.

Consequently, both the theoretical advancements leading to the ADC model in combination with the practical requirements to assess the current work situation in a more comprehensive way have led to the need to further develop the JCQ to the JCQ 2.0. This is in line with Johnson’s [11] call for future DCS area development in which “… at the very least, the operationalization and measurement of the three core dimensions needs to be fundamentally modified and updated” (p. 19). The JCQ 2.0 has evolved as an instrument to address these limitations.

Agbenyikey et al. [in this Special Issue; see Appendix A, A.2] describe both the multi-stage development process of the JCQ 2.0 as well as its scales and its psychometric characteristics.

So far, the fundamental concepts and the structure of the JCQ 2.0 have not yet been verified. Therefore, the aim of our paper is to test whether the theoretical constructs of D, C, and S-S at both the task and the organizational level and their interrelations can be verified with empirical data. More specifically, the paper aims to test whether the structure of the relationship between D, C, and S-S remains consistent (a) after the addition of new scales for all constructs D, C, and S-S, (b) across both the task and the organizational level, and (c) across countries with different contexts (e.g., culture, national policy, union density) in as far as this is possible with the data available.

Demonstrating distinguishable constructs for D, C, and S-S at both the task and the organizational level with specific indicators for each construct will allow for aggregating scores into composites, facilitating further testing and comprehensive communication of findings as presented by Formazin et al. [in this Special Issue, see Appendix A, A.3], and better informing practical outcomes in the future.

In the following, we will give a brief description of the ADC model (see Section 1.1) and then focus on D, C, and S-S constructs at both the task and the organizational level as well as the scales of the JCQ 2.0 that assess these constructs (see Section 1.2). Based on this information, we will present our research hypotheses (see Section 1.3) and describe the specific characteristics of the four studies we have conducted (see Section 1.4).

### 1.1. The Associationalist Demand/Control Model

The ADC model expands the existing task-level focused DCS model to provide a theoretical platform for a “multilevel” structure. It incorporates perspectives from psychology, sociology, public health and organizational science as described in detail by Karasek et al. [in this Special Issue, see Appendix A, A.1]. The theoretical meaning of the scales when explained in a multilevel context is consistent with the classic general meaning implied by the D, C, and S constructs [1,2,3,12]. This gives us a theoretical basis for empirically testing new multilevel D, C, and S-S scales with a narrative familiar to researchers when extending the constructs across multiple levels at work. Considering the different levels can help inform workplace policies that aim at enhancing employees’ working conditions and reducing stress factors: In order to achieve a stable effect of workplace interventions, problems need to be tackled at their roots.

In the following, the constructs of demand, control, and stability-support and their respective scales on the task as well as on the organizational level—based on the ADC model [see Appendix A, A.1])—will be defined. A detailed description of the scales is presented by Agbenyikey et al. [this Special Issue, see Appendix A, A.2].

### 1.2. Demand, Control, and Stability-Support in the JCQ 2.0 on Both Levels

#### 1.2.1. Demand Scale Definitions in the JCQ 2.0 on Both Levels

In the context of the JCQ 2.0, it is the individual’s job that is the source of the demands for resources and inputs. The job is the employee’s skill application area and is where expected “output” is delivered [for details, see Appendix A, A.1].

In relation to job task demands, in the JCQ 2.0, there are three scales: “quantitative demands”, “emotional demands”, and “physical demands”. The first is a revision of the established scale “psychological demands” [1,12] from the JCQ 1 while “emotional demands” is a new scale with special relevance to workers who have to deal with other people at work [13]. These two are assumed to be empirically closely related and will be considered in the following. The scale “physical demands” was excluded from analyses because it represents a different basic explanatory framework [12].

In relation to organizational level demands, two JCQ 2.0 scales are specified: “organizational reconstruction” refers to adaptive changes in the organizational structure considered stressful for employees while “organizational disorder” refers to the degree to which organizational instability is causing its own set of “job demands” for employees, and thus is associated with extra load on the workers that they consider unnecessary [see Appendix A, A.1 and A.2].

#### 1.2.2. Control Scale Definitions in the JCQ 2.0 on Both Levels

In the context of the JCQ 2.0, control refers to the strategies individuals have developed to maintain their stability and to grow in the context of work. To get their tasks done, individuals must exert control over and within their environment in order to optimally process and utilize their input resources. Skills—which are a sub-dimension of control—are the capabilities developed by workers to successfully deal with environmental challenges inside the organization (see Appendix A, A.1 for further details).

At the task level, three JCQ 2.0 scales assess control. The two scales “decision authority” and “skill discretion” both stem from the classic DC model [1,12] and have been part of the JCQ 1 as well. “Conducive development” is a new scale that focuses on organizational facilitation of an employee’s ability development in accordance with the Conducive Economy concept [14] and is related to skill discretion; it can be considered “updating” the established concept of skill discretion in line with the current global economic context.

At the JCQ 2.0 organizational level, there are three scales that assess control (see Appendix A, A.1). The scale “organizational decision latitude” reflects employee input into decision processes [15] while “procedural justice” reflects fairness in the decision-making processes [16]. “Conducive communication” goes a step further in the high control direction and captures aspects of innovative organizational communication which supports co-partnering between employees and customers while considering customers’ needs according to the Conducive Economy concept [14].

#### 1.2.3. Stability-Support Scale Definitions in the JCQ 2.0 on Both Levels

The buffering effects of social support so far discussed in the context of DC job stress research [17] can be understood more broadly based on the ADC Model: social support structures provide assistance, offer channels for effective organizational contribution, and protect the worker’s daily stability from the environment’s volatility, thus supporting decision latitude as a regulatory construct [see Appendix A, A.1]. With a focus on the balance between the challenges posed by the environment and the responses required by the individual, social groups in the workplace and workers’ supervisors can provide a stable basis of action for workers. Hence, the concept is relabeled stability-support (S-S).

At the task level, to assess social stability-support, the JCQ 2.0 retains modified versions of the two original JCQ 1 scales “supervisor support” and “co-worker support”. Both continue to reflect instrumental as well as affective aspects of social support and contain an additional item about respect from the supervisor and the co-workers, respectively, reflecting a close relation between the stability-support scales and Siegrist’s effort–reward imbalance (ERI) model [18]. Co-worker support is conceptualized as “people one works with”, making it broader than just colleagues (e.g., including suppliers or business partners). “Collective control” is a new scale that relates to collective forms of control among colleagues through the development of work groups [2]. “Negative acts” is a short, summary-level measure of adverse aspects of social relations at work including social isolation and bullying and can be considered a negative form of social support [19].

JCQ 2.0 organizational stability-social support is assessed by three scales: “organizational rewards”, “consideration of workers’ interests” and “psychosocial safety climate”. The first scale is based on the aspect of rewards in the form of money and appreciation of Siegrist’s ERI-model [18] while the second scale extends this to the importance of employees’ wellbeing in the face of organizational changes [see Appendix A, A.1]. The third scale derives from Dollard’s PSC model and assesses priorities and systems for the protection of worker psychological health and safety [20].

#### 1.2.4. Summarizing the Differences Between the JCQ 1.7 and the JCQ 2.0

Comparing the established JCQ 1.7 with the newly developed JCQ 2.0, one can state that the latter assesses the psychosocial work environment in a much wider way than the former. The JCQ 2.0 contains ten scales on the task level—only five of them are revised JCQ 1.7 scales, one has been kept unchanged while two are literature-based and two have been newly defined and developed. Moreover, while no organizational level scales were available in the JCQ 1.7, the JCQ 2.0 includes eight: three of them have been derived from literature while the other five have been newly constructed from theory.

Because the JCQ instrument has been revised so profoundly, it requires a thorough psychometric evaluation—which is presented by Agbenyikey et al. [Appendix A, A.2]—as well as a replication of its underlying structure which is the focus of this manuscript. In the following, we will present which hypotheses we wish to test with our study with regard to the JCQ 2.0’s underlying structure.

### 1.3. Research Hypotheses

Based on the ADC model as presented by Karasek et al. [see Appendix A, A.1] and the JCQ 2.0 as described by Agbenyikey et al. [see Appendix A, A.2], we propose the following hypotheses regarding the JCQ 2.0’s structure:

**Hypothesis 1a.**  *On the task level, there is a three-factor structure wherein each dimension of D, C, and S-S will be represented as one factor.*

**Hypothesis 1b.**  *Each factor will have indicators according to the theoretical considerations in Section 1.2:*

- *D: quantitative demands and emotional demands;*
- *C: skill discretion, decision authority, and conducive development;*
- *S-S: supervisor support, co-worker support, collective control, and negative acts.*

**Hypothesis 1c.**  *Both C and S-S will be positively interrelated to a substantial degree whereas their associations with D will be of a small magnitude.*

We assume that the newly developed scales will allow us to model the three factors in a broader way without altering the three factors’ meaning due to their theoretical subsumption according to the ADC Model [see Appendix A, A.1]. Regarding the association of the three factors, the expected substantial relation between C and S-S is not only in line with the ADC Model but also with the Job Demand Resources Model [21,22,23,24] which considers aspects of both C and S-S as job resources. Moreover, past empirical results indicate a positive association [5,6,25,26,27,28,29,30]. Associations of C and S-S with D have been somewhat mixed in the past but are mainly of a small magnitude for D and C [5,6,25,26,27,30,31] and ranging from zero to a small negative magnitude for D and S-S [5,6,25,26,27].

**Hypothesis 2a.**  *Similar to the task level, there is a three-factor structure at the organizational level in which each dimension of D, C, and S-S is represented as one factor.*

**Hypothesis 2b.**  *Each factor has indicators according to the theoretical considerations in Section 1.2:*

- *D: organizational disorder and organizational reconstruction;*
- *C: organizational decision latitude, procedural justice, and conducive communication;*
- *S-S: rewards, psychosocial safety climate, and consideration of workers’ interests.*

**Hypothesis 2c.**  *Both C and S-S are strongly positively interrelated whereas their associations with D are of a smaller and negative magnitude.*

Because the organization is more distal to the workers than their tasks, it can be assumed that workers find it more difficult to differentiate between aspects of the organization than between aspects of the task, leading to higher associations among organizational level factors. Bakker et al. [24] describe consensus among employees regarding job characteristics at a level higher than the task level. In addition, the characteristics on the organizational level can be assumed to be more similar as is reflected in the concept of organizational culture [32].

**Hypothesis 3.**  *In a structural model, the three factors for D, C, and S-S at the organizational level are antecedents to the three task level factors for D, C, and S-S, i.e., the organizational level factors are exogenous factors and the task level factors are endogenous factors.*

According to the ADC Model [see Appendix A, A.1], the work characteristics at the organizational level are the framework in which work at the task level is embedded. This is also in line with Multilevel JD-R Theory which assumes that “employees are nested in teams, which, in turn, are nested in organizations” (p. 38, [24]).

### 1.4. The JCQ 2.0 Pilot Studies

The JCQ 2.0 development process was undertaken by an international group of researchers, among them researchers from Korea, China, Australia, and Germany. We followed a stepwise development procedure which is described by Karasek et al. and Agbenyikey et al. [in this Special Issue; see Appendix A, A.1 and A.2]. The first study was undertaken in Korea, followed by China, Australia, and Germany. In the first pilot studies, not all scales and items of the JCQ 2.0 could be included because of the ongoing development processes: there were regular workshops in which further items and scales were developed. Table 1 gives an overview of which scales were available in which pilot study and how many items each scale contained. As can be seen from Table 1, the number of scales and items rose from the first pilot study in Korea to the last pilot study in Germany. This has consequences for the models that could be established: Hypotheses 1a to 1c could be tested over all four pilot studies albeit no multigroup models could be established because of the differing scales available per country. We note that this aspect warrants consideration in the discussion of our results regarding the generalizability of our results. In contrast, Hypotheses 2a to 2c as well as 3 could only be tested in the German sample because this—as the latest pilot study—was the only one that included all relevant scales on both the task and the organizational level. Hence, our results regarding the organizational level will only allow for preliminary conclusions.

## 2. Method

### 2.1. Data Collection, Translation Process, and Data Handling

Data were collected in Korea, China, Australia, and Germany, addressing both cross-country and systemic differences in workplace stress and thereby taking a global worker-health perspective into account. Details on data collection of this multi-country design, the translation process, the pre-testing, handling of missing data, and scale construction are described by Agbenyikey et al. [this Special Issue, see Appendix A, A.2]. In this paper, we restrict our focus to confirmatory factor analyses and structural equation modeling of the JCQ 2.0 scales.

### 2.2. Confirmatory Factor Analyses

Confirmatory factor analyses were based on the JCQ 2.0 scale scores. For confirmatory factor analyses, the 10 imputed datasets per country were used as input. This allowed us to use the same database for all analyses of the data as presented in the other papers of this Special Issue [see Appendix A, A.2 to A.5]. The software used—Mplus 7.31 [33]—combined results from the 10 datasets and accounted for the fact that data has been imputed when calculating the chi-square value [34].

At the task level, based on theoretical considerations, a model with three correlated factors was established (model 1). In the German sample, the factor for demand (D) had two indicators, the factor for control (C) had three indicators, and the factor for stability-support (S-S) had four indicators, with each indicator loading on one factor only (model G1). Depending on available scales per country, the factors partly had fewer indicators in the other three datasets (models K1, C1, and A1), for details, see Table 1. Since the number of indicators differed between the four countries, it was not possible to meaningfully establish a combined model over all four countries and to explicitly test for measurement invariance. We hence assessed similarity across countries by establishing comparable models in each country, i.e., with three underlying factors (according to Hypothesis 1a) which are all correlated (according to Hypothesis 1c) and indicators as theoretically expected according to Hypothesis 1b.

Based on empirical results in the German data, an alternative model (model G2) was derived; this revised model was then replicated in the other three datasets in addition to the original model (models A2, C2, and K2). This methodological rigor in validating the JCQ 2.0 was important to ensure its applicability in diverse settings across different countries.

At the organizational level, modeling the structure of the JCQ was only possible in the German dataset which contained enough indicators. Hence, all these analyses were restricted to the German dataset. Analogous to the task level, a three-factor-model was established at the organizational level with two (D) or three (C, S-S) indicators per factor, again with each indicator loading on one factor only.

In a final model on the structure of the JCQ 2.0, both the task level model and the organizational level model were combined, again only based on the data of the German sample. The organizational level factors were modeled as antecedents to the factors on the task level, assuming that the organizational factors influenced the factors on the task level. Each factor on the task level was regressed on its corresponding factor at the organizational level, allowing for no correlation among the task level factors and assuming that their interrelations were fully explained by the organizational level factors. Before establishing this model, the variance inflation factor (VIF) for each indicator was estimated via SPSS 29 to identify any possible extreme collinearity [35]. Moreover, average variance extracted (AVE) was assessed as the average of the squared standardized pattern coefficients [35].

The following fit indices, differentially sensitive to deviations from the applied model, were considered to decide how well a model fitted the data [36]: the Chi-Square-Value χ^2^, the Comparative Fit Index *CFI*, the Root Mean Square Error of Approximation *RMSEA* and the standardized root mean square residual *SRMR*. Note that the χ^2^-value is sensitive to sample size: even when deviances between model implied and observed covariances are small, the model will be rejected in small samples. The *CFI* is an indicator of relative model fit that can attain values of 0 ≤ *CFI* ≤ 1; model fit is regarded as acceptable if *CFI* ≥ 0.90 [37]. *RMSEA* values less than 0.08 imply acceptable model fit [38] as do values of *SRMR* < 0.11 [36]. When judging model fit, one should bear in mind that these cut-off values are no “golden rules”.

## 3. Results

### 3.1. Description of the Sample

The pilot in Korea was conducted among transit workers in Seoul while the pilot in China was a community-based random sample survey of employees from Kunming. In Australia, data were collected via the random population based Australian Workplace Barometer interview survey among employees from four Australian states and two territories. Finally, the German pilot was a population-based random survey in the Ruhr area of Germany. Sample sizes are depicted in Table 2. The mean age of participants varied between 42.1 years (SD = 3.9) in Korea and 45.7 years (SD = 12.2) in Australia. A full description of the four samples from Germany, Australia, Korea, and China can be found in Agbenyikey et al. [Appendix A, A.2].

### 3.2. Confirmatory Factor Analyses—Task Level

As the German data was the most comprehensive, analyses were conducted on the German data and replicated—where possible—with the Korean, Chinese, and Australian data.

**Figure 1:**
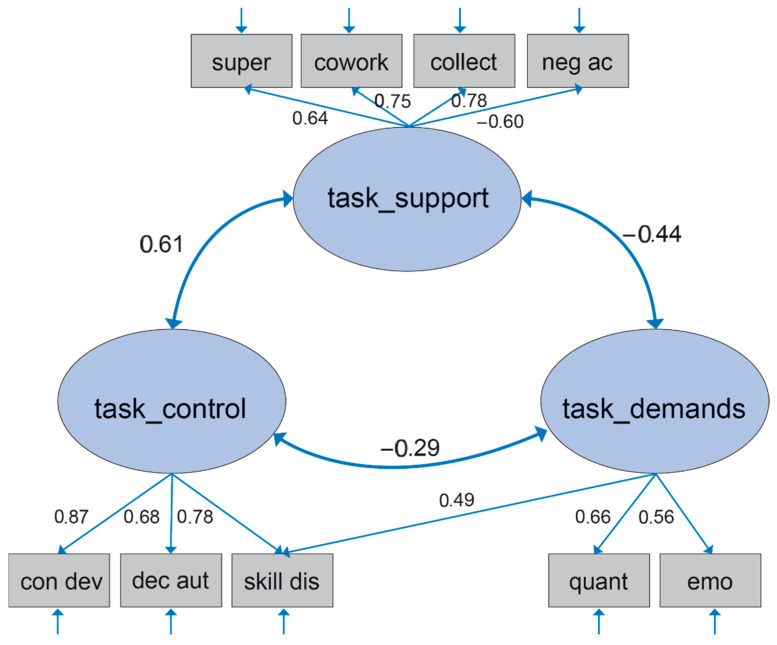
Structure of the JCQ task level—German data. Note: all factor loadings are standardized factor loadings λ; for all λ: *p* ≤ 0.05.

The model “G1” with three correlated factors on the task level had low fit with regard to all fit indices (see Table 2). A striking result in this model was the high residual correlation between the C-indicator “skill discretion” and the two D-indicators “quantitative demands” and “emotional demands”. Modeling this association by allowing an additional path from D to “skill discretion” in a slightly revised model G2 led to a rise in model fit (see Table 2). In this model, all factor loadings were of substantial and positive magnitude with the only negative loading for “negative acts” as expected—as can be seen in Table 3. There was a strong positive association between control and stability-support. Both C and S-S were negatively related to the factor for demands. A graphical presentation of the model is depicted in Figure 1. In the following, model G2 was considered the final model.

Similar models—i.e., with each scale loading on only one factor as was the case for model G1—were established in the other three datasets as models K1, C1, and A1; fit indices are presented in Table 2. In Korea, China, and Australia, for both C and S-S there was one indicator less than in the German data as the scales for “conducive development” and “negative acts” were not used in these three samples (see Table 1). In addition, the scale “collective control” was not used in Australia (see Table 1).

In both Korea and China, it was not possible to establish a model with three correlated factors with each indicator loading on one factor only, i.e., models “K1” and “C1”, respectively. For model “K1”, the chi-square value could not be estimated, possibly indicating that the structure was too strict [Bengt O. Muthen, Mplus discussion forum, available at http://www.statmodel.com/discussion/messages/9/61.html?1465397517 (accessed on 6 June 2016)]. For model “C1”, no fit indices were estimated because for one imputed dataset, the residual covariance matrix was not “positive definite”, leading to a standardized factor loading λ > 1—a so-called Heywood case. Additionally, another imputed dataset did not converge. The model with each indicator loading on one factor could only be established in Australia as model “A1”; however, the RMSEA index implied low fit (see Table 2).

Despite the fact that two or three scales were missing in the three datasets from Korea, China, and Australia, it was possible to establish a model with three correlated factors for demand, control, and stability-support, respectively, with one additional loading from the scale “skill discretion” on the demand factor for each dataset, i.e., model “K2” for Korea, model “C2” for China, and model “A2” for Australia. Model fit was at least acceptable in each country. Hence, these models K2, C2, and A2 were considered the final models. The scales loaded on the factors as expected, factor loadings were of a medium-to-large magnitude in all countries as can be seen in Table 3. An exception was the unexpected factor loading for “skill discretion” on the factor for demand with a value ranging between 0.17 ≤ λ ≤ 0.43 that could, however, be shown in all four datasets. The correlation between the latent factors for control and stability-support, respectively, was similar and of a medium-to-large magnitude across countries. This was not the case for the other two factor correlations: while the correlation between demand and control was of a substantial negative magnitude in Korea, it was of a smaller negative magnitude in Germany and Australia, respectively, and unrelated in China. The latent factors for demand and stability-support were not correlated in China and Korea and correlated negatively in Australia and Germany.

### 3.3. Confirmatory Factor Analyses—Organizational Level

The three-factor-model at the organizational level with two (D) or three (C, S-S) indicators per factor could only be modelled with the German data. It had satisfying fit with χ^2^ (17, *N* = 2326) = 134.64; *p* < 0.01; *CFI* = 0.971; *RMSEA* = 0.055; *SRMR* = 0.031. However, there was a residual correlation between two of the three indicators of the control factor: “organizational decision latitude” and “procedural justice”.

**Figure 2:**
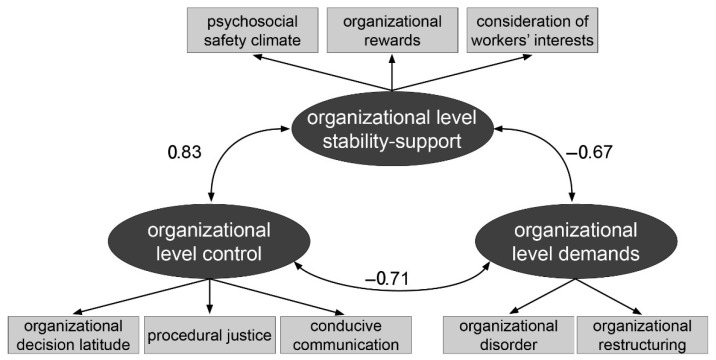
Simplified structure of the JCQ organizational level—German data. *Note*: for reasons of easier presentation, values for factor correlations, factor loadings, and residual variances are only shown in Figure A1 in Appendix B.

**Figure A1:**
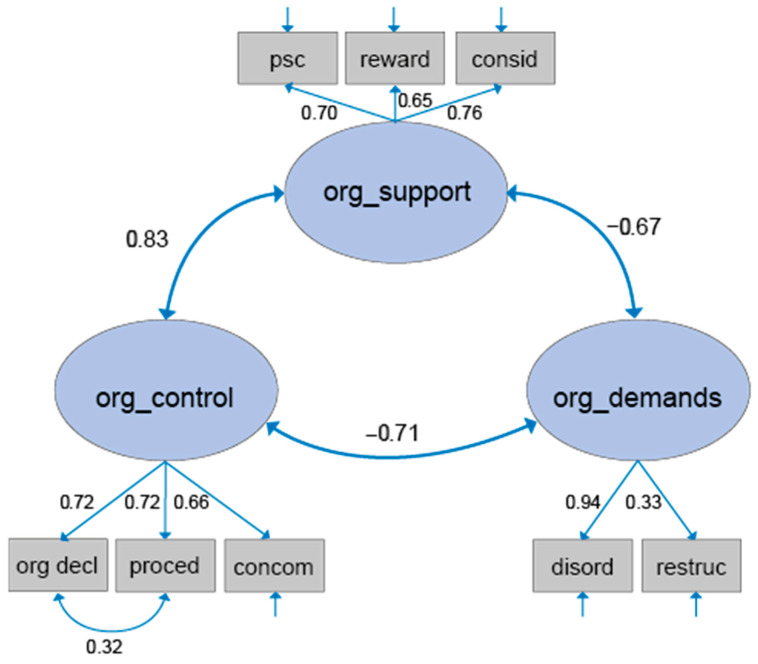
Structure of the JCQ organizational level with factor loadings and residual variances and correlations—German data. Note: all factor loadings are standardized factor loadings λ; for all λ: *p* ≤ 0.05.

Accounting for this association by modeling an additional residual correlation led to a model with good fit: χ^2^ (16, *N* = 2326) = 89.85; *p* < 0.01; *CFI* = 0.982; *RMSEA* = 0.045; *SRMR* = 0.023. In this model, all indicators loaded on their respective factors with loadings varying between 0.65 ≤ λ ≤ 0.94, with one exception: the factor loading of “organizational reconstruction”, the sole single-item measure, had a value of λ = 0.33. The residual correlation was estimated at ρ_resid_ = 0.32. The model—which we accepted as the final model for the organizational level—is depicted in a simplified manner in Figure 2; the complete model is presented in Figure A1.

At the organizational level, the correlation between control and stability-support was strong and positive with a value of ρ = 0.83 whereas both factors correlated negatively and to a lower degree with the demand factor with ρ = −0.71 and ρ = −0.67, respectively. All three associations on the organizational level were of a higher magnitude than those on the task level.

### 3.4. Confirmatory Factor Analyses—Combining the Task and Organizational Level

Again, the model combining the task and the organizational level could only be established in the German dataset due to data availability. The variance inflation factors (VIF) for all indicators were low, mainly ranging between 1 and 2 and with the highest value of VIF = 2.501, implying no extreme collinearity [35]. The combined structural model had model G2 for the task level and the model with the residual correlation on the organizational level. Furthermore, the organizational level factors were modeled as antecedents to the factors on the task level. Fit of this model was satisfactory with χ^2^ (111, *N* = 2326) = 1273.24; *p* < 0.01; *CFI* = 0.908; *RMSEA* = 0.067; *SRMR* = 0.049, with all indicators loading on their respective factors. There was one striking point though: “supervisor support”, modeled as an indicator of task level stability-support, had substantial residual correlations with the three indicators of the organizational level stability-support factor.

**Figure 3:**
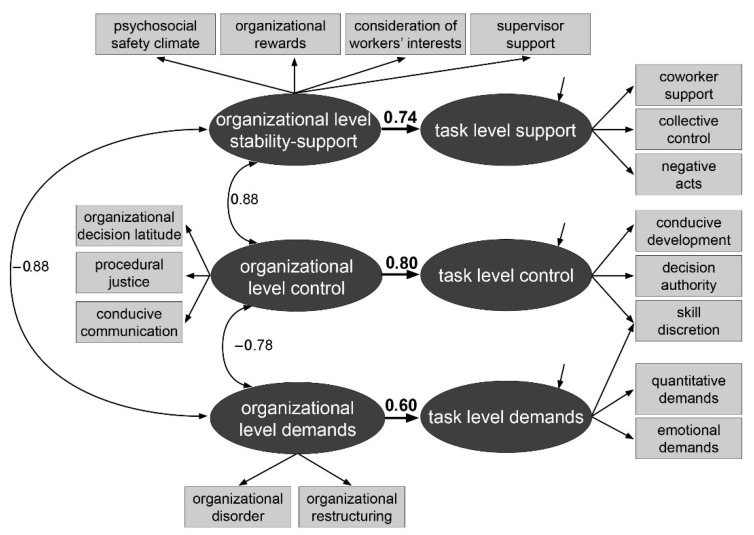
Simplified structure of the JCQ combining both levels—German data. *Note*: for reasons of easier presentation, values for factor correlations, factor loadings, and residual variances are only shown in Figure A2 in Appendix B.

**Figure A2:**
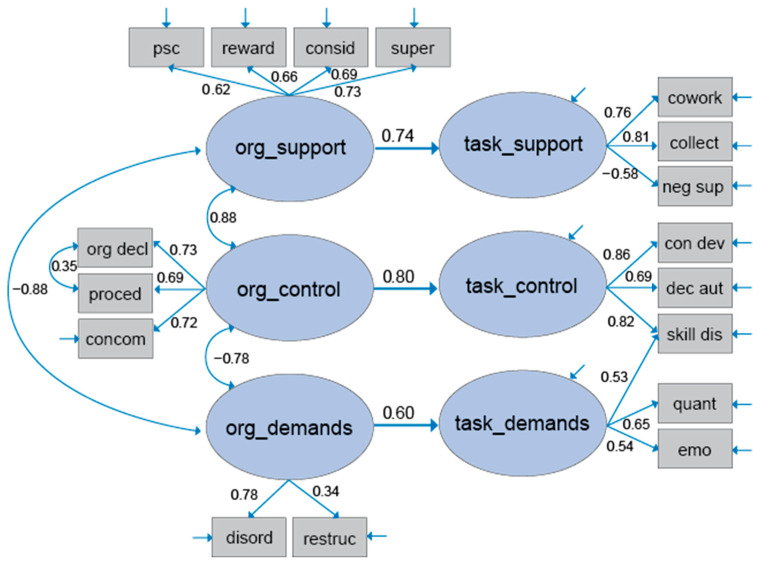
Structure of the JCQ combining both levels with factor loadings and residual variances and correlations—German data. *Note*: all factor loadings are standardized factor loadings λ; for all λ: *p* ≤ 0.05.

Altering the original structure by modeling “supervisor support” as the fourth indicator of organizational level stability-support, leaving the task level stability-support factor with three indicators, led to a better model fit. This model in a simplified manner is depicted in Figure 3; the complete model is presented in Figure A2.

In this final structural model with χ^2^ (111, *N* = 2326) = 1029.49; *p* < 0.01; *CFI* = 0.927; *RMSEA* = 0.060; *SRMR* = 0.043, factor loadings were substantial for all indicators with values close to those in the two separate models, ranging between |0.34| ≤ λ ≤ |0.86|. “Skill discretion” was the only indicator with loadings on two factors whereas all other indicators loaded on one factor only. The residual correlation between “organizational decision latitude” and “procedural justice” remained at a medium value with ρ_resid_ = 0.35. AVE ranged between 0.33 for task level demands and 0.63 for task level control.

The beta coefficients from the organizational to the task level factors were of a high value, the lowest was β = 0.60 for the demand factors and the highest was β = 0.80 for the control factors. Associations on the organizational level were of a high magnitude, too: the association was positive for organizational control and organizational stability-support with ρ = 0.88; the correlations of each factor with the organizational demand factor were negative with ρ = −0.78 and ρ = −0.88, respectively.

## 4. Discussion

This is the first study to examine the structure of the DCS-S-model on both the task and the organizational level as proposed by ADC theory [see Appendix A, A.1]. Identifying the factors underlying the JCQ 2.0 is the basis for showing—in a next step—how they are linked to relevant health outcomes as is presented by Formazin et al. [Appendix A, A.3]. This, in turn, will allow for the development of workplace interventions targeting stress reduction and disease prevention in the future.

Our aim was to show that the theoretical constructs of D, C, and S-S on both the task and the organizational level and their interrelations are consistent with empirical data. Results of our analyses largely supported our hypotheses about three correlated factors on the task (Hypothesis 1a) and organizational levels (Hypothesis 2a) with the organizational level factors as antecedents to the task level factors (Hypothesis 3).

In the following, we will elaborate on how well the empirical results supported our hypotheses as proposed in Section 1.3.

### 4.1. Structure on the Task Level

With regard to the task level, we found that it was possible to establish a three-factor structure in which each dimension of D, C, and S-S was represented as one factor. This factorial structure was the same across all four countries considered in our analyses, confirming Hypothesis 1a.

All scales functioned as indicators of the underlying factors as expected with one exception: there was a high relation between the scale skill discretion and the factor for demand. Model fit was acceptable when an additional loading from the factor “demands” to the indicator “skill discretion” was modeled. This additional loading could be shown consistently across all four countries, implying that this was not a spurious association. We can hence not fully confirm Hypothesis 1b that each factor has indicators derived from the ADC model. It must be noted that the loading of skill discretion on the factor for control was higher than on the factor for demand across all four pilot studies—as classically predicted. Nevertheless, the consistency of this unexpected result is important because of its implications relating to the evolution of the relationship between skill as a demand or skill as an indicator of potential control.

Why did this additional loading occur? It is unlikely that this result was due to a psychometrically weak scale “skill discretion” because the latter did not include the item on “repetitive work” any longer. That item has shown contradictory relations with other skill discretion items in the past ([25,26,28,29,30,31,39,40,41,42], (Appendix A, A.2)).

Rather, it is likely that this result was due to the wording of the items included in the scale skill discretion—they start with “my job requires me”. This is a wording that can indicate a demand. Consequently, a very high level of skill discretion could be considered a demand [43]. This would be in line with a Japanese study suggesting that “skill discretion could be a source of qualitative or intellectual job demands independently of quantitative demands” (p. 371) [29] due to rapid technological changes in the past and that “intellectual and quantitative demands might require separate measurements” (p. 371) [29]. In a study on women working in nursery schools, the items from the scales psychological demands and skill discretion loaded on one factor in exploratory factor analysis [44]. It has also been shown that the two scales “skill discretion” and “decision authority” had different effects on health outcomes [45,46,47]. Therefore, it seems warranted to investigate this aspect in the future; cognitive interviews with workers from different branches could be a promising way to gain a deeper insight.

Compared to the established scale “skill discretion” [30,44], the new JCQ 2.0 scale “conducive development” was advantageous in that it considered the context of skill development. In addition, the conducive development items did not include the word “requires” that could be misunderstood as a demand. Instead, they assessed real on-the-job learning that is assumed to be distinct from “competence demands”, the latter described with items like “I am expected to develop my competence” or “I feel pressure to continually learn in order to manage my work task” [48]. Empirical results on the scale “conducive development” so far imply that it has been designed in a clear and less ambiguous way, rendering it a useful scale for further research on task level control.

Turning to Hypothesis 1c, on the task level, a strong association between the factors for control and stability-support could be observed across all four pilots. These positive and high correlations based on latent factors were in line with expectations of ADC-theory [Appendix A, A.1], past results mainly based on scale correlations [5,6,25,26,27,28,29] and with the job demand–resources model which considers both job control and social support as resources [21,22,23,24]. This strong relation has been taken up in the scale “collective control” which is a synthesis of the two concepts of job control and social support [2], modeled as an indicator of social stability-support, and for which Choi et al. [49] have shown a partial synergistic interaction effect on general psychological distress in men and a full one in women. In terms of practical implications, one would hence expect interventions that raise aspects of both stability-support and control at the task-level, e.g., by granting work groups the decision latitude to distribute their tasks among each other autonomously, to strongly promote workers’ health and wellbeing.

In contrast to the very clear association between task level control and stability-support, their correlations with the factor for demand were not as homogenous across the countries.

The correlation between demand and control differed: There was no association in China, it was of small negative magnitude in Australia and Germany with ρ = −0.15 and ρ = −0.29, respectively, and there was a substantial negative association in Korea with ρ = −0.53. It is possible that the high negative association in Korea was due to the specific sample—it only included workers from one very large urban transit company. In contrast, in China, Australia, and Germany, participants were from a wide range of occupations and companies.

At the same time, one has to bear in mind that in the past, results on the association between demand—restricted to quantitative demands only—and control have been mixed as well. Positive associations have been reported with *r* = 0.14 in the French GAZEL study [26], *r* = 0.18 in a Canadian hospital sample [31], *r* = 0.40 in Swedish workers [28], and *r* = 0.31 for men and *r* = 0.45 for women in a Japanese study [29]. In contrast, Mase et al. [44] found demands and skill discretion items to load on a common factor in exploratory factor analysis. A study considering six different samples found results varying between −0.04 ≤ *r* ≤ 0.35 for men and −0.14 ≤ *r* ≤ 0.45 for women, with the highest values from the Japanese sample and lower values in the samples from Canada, the USA, and the Netherlands [50]. In meta-analyses, the demand–control relationship was found to be very near zero overall [5,6] as was the case in an US American sample [27] and a Chinese sample [25]. For future research, it would be worthwhile to consider additional data sources, e.g., expert ratings of working conditions, qualitative data based on interviews with workers or structural information on economies in different countries, to shed further light onto this question.

Finally, the third association between the factors for demand and stability-support was virtually zero in the two Asian countries, China and Korea, whereas it was of medium size and negative value in the two Western countries, Australia and Germany. Other researchers have reported differing results as well. In some samples, the association was negative [5,6,26,27,28,30], in other samples, it was close to zero [25]. It is possible that this result was due to cultural differences; however, it was not possible to answer this question with our research design.

Thus, it was not possible to draw a final conclusion regarding Hypothesis 1c and more cross-country research is needed in the future [32]. Our four pilots showed partial international consistency at the task level while also suggesting a particular focus for further research. First, the control–stability-support associations were consistently positive. Second, the demand–control associations tended to be mainly low, if inconsistent as has been reported in the literature. Third, we observed a substantial difference in the associations between Demand and Stability-Support, with the associations negative in the Western countries Australia and Germany, but zero in the Eastern countries Korea and China. More contextual information will be necessary for figuring out the nature of the associations, in particular between D and S-S, and between D and C, while at the same time making sure that the concepts are assessed in a similar way across countries. It may be hard to capture this information solely in quantitative analyses. Instead, in-depth contextual analyses derived from qualitative studies are required.

### 4.2. Structure on the Organizational Level

Turning to the structure on the organizational level—only testable in the German sample—it was equivalent to the structure on the task level. Three factors for demand, control, and stability-support could be established with indicators as expected, confirming Hypothesis 2a. The three factors were correlated to a high degree, confirming Hypothesis 2c, and the magnitude of associations on the organizational level was higher than on the task level.

It is possible that this result was due to the perspective of the workers: the organizations’ characteristics were described from their point of view. Bakker et al. [24] have postulated that there is consensus regarding job characteristics among individuals working together. In addition, it might have been more difficult for workers to differentiate between aspects of the organization than between aspects of the task for reasons of lower (the organization) vs. higher (their task) proximity. With the available data, it was not possible to test this interpretation; this would have required data from organizations assessed by management or by external experts which is a direction of further research.

Moreover, one cannot rule out that characteristics on the organizational level were truly more similar as is reflected in the concept of organizational culture [32]. Hence, organizational culture may have affected all organizational level scales in a similar way. Again, this points to a direction of further research.

With regard to the third hypotheses for the organizational level, in our model, each scale served as an indicator of one of the three factors. For the factor demand on the organizational level, there were only two indicators available of which one was a single item, not a scale, because additional items were not applied in the German study. For this factor, the factor loading was close to unity for “organizational disorder”, the scale score indicator, and very low for “organizational reconstruction”, the single item indicator. These empirical results imply that additional items are required for the scale “organizational reconstruction”. Presumably, with two scale scores as indicators that are metric in nature, the factor loadings will become more similar, rendering the model more stable.

A further aspect needs to be mentioned. It was necessary to model an additional residual correlation between two indicators for organizational control: organizational decision latitude and procedural justice. This might have been due to the fact that both scales used items that tapped similar aspects. Whether this residual correlation was specific to the sample used or a general result awaits replication in other datasets. Available evidence points in the second direction [51,52]. Considering this aspect, one can summarize that Hypothesis 2b was nearly fully confirmed.

One has to emphasize that only the German dataset contained enough indicators necessary to establish a structural model on the organizational level. It was hence not possible to replicate the structure established in the German dataset in the other three datasets. Consequently, the structure on the organizational level could only be affirmed preliminarily and requires further data collection and analyses for further confirmation.

### 4.3. Structure on Both Levels

Finally, turning to the structural model considering both the task and the organizational level, solely based on the German data, the latter was confirmed as proposed: there were three factors for demand, control, and stability-support on each level. Each task level factor was associated with its compatible organizational level factor, and the associations were of a high magnitude. The interrelations between the three factors on the task level were fully explained by their associations with their respective organizational level factors and the interrelations among the latter, confirming Hypothesis 3. The factor loadings for the indicators in the combined model were similar to those in the two separate models that only considered the task and the organizational level, respectively.

However, in the final model, “supervisor support” had to be modeled as an indicator of organizational level stability-support instead of task level stability-support in order to achieve model fit. Our interpretation of this unexpected result is that it was presumably due to the data assessed via workers’ self-report. Through the workers’ eyes, the supervisors were located at the level of the organization, not at the level of the task, possibly because they were seen as part of the organization’s management. It will be worthwhile to further investigate this aspect in the future by applying qualitative methods, e.g., cognitive interviews.

The full model could only be established based on the German data because in the other three datasets there were not enough organizational level indicators available required for establishing a stable model as discussed in Section 4.2. Hence, the full model needs to be replicated in future studies. This especially pertains to supervisor support being an indicator of organizational level stability-support. Here, a triangulation of questionnaire-based data with, for example, qualitative data is a promising next step. It should be noted, however, that correlations between supervisor support and scale scores of organizational stability-support available in the Australian data, e.g., rewards and psychosocial safety climate, were of substantial magnitude with *r* = 0.38 and *r* = 0.47, respectively [Appendix A, A.2], indicating that the association found in the German data was not spurious.

While considering the preliminary nature of our results, they imply that interventions tackling working conditions at the organizational level will have an influence on the working conditions at the task level. This is in line with a conceptualization of trickle-down effects from the organizational level via the team level to the individual level [24] and empirical results implying that improving the work organization can help to decrease bullying in workplaces [53]. Both task-level and organizational-level factors can guide interventions to improve mental health in the workplace and prevent chronic conditions [24], i.e., actions to raise organizational decision latitude on a higher organizational level will presumably lead to higher decision authority on the workers’ task level.

### 4.4. Strengths and Limitations

This is the first paper to test the structure of the DCS-S-model based on the ADC Model [Appendix A, A.1] and the revised JCQ 2.0 [Appendix A, A.2]. This cross-country validation of the JCQ 2.0 supports the instrument’s utility, albeit currently limited to the task level. Such a cross-country confirmation itself is unusual: research testing whether differences between countries, e.g., due to national policy, shape organizational behavioral differences between countries is relatively rare, mainly because most studies are based on nationally homogenous studies [32].

Nevertheless, there are some limitations to our results that need to be considered.

While the structure of D, C, and S-S on the task level could be tested in all four datasets, only the German dataset allowed both testing of the model’s structure on the organizational level and combining the task and the organizational level in a single model. Consequently, more studies are needed which robustly assess the work environment with sufficient scales to allow establishing a model that considers both levels, i.e., the task and the organizational level, together. Moreover, further studies are necessary across different countries to investigate whether the associations between the factors vary systematically between countries. Assessing the same items and scales across different countries will allow multigroup measurement invariance testing which, in turn, will strengthen conclusions regarding cross-national equivalence. By additionally employing cognitive interviews, it will be possible to further investigate whether workers perceive demand, control, and stability-support differently due to, e.g., cultural differences.

Analyses in this paper were based on scale scores of the JCQ 2.0, not on single items. Due to the high number of items in the JCQ 2.0 (e.g., 49 items in the German task and organization level version, plus additional external-to-work items [Appendix A, A.4]) and simultaneously a low number of items per scale (most often, only two to three items per scale), a model based on single items would have been difficult to establish and would have contained a risk of low stability. In addition, the varying number of scales per country and of items per scale across the countries would have led to low comparability of results. Since our aim was to develop an instrument that will allow to assess the working conditions on three levels, we strived for comparably short scales in order to limit the burden on participants in our study when answering all items.

Scales with more items would have most likely had even higher internal consistency, leading to higher AVE for the underlying factors, and allowed for even more complex models than the ones we have established. However, we believe that keeping the instrument as focused as possible with as few items as possible allows for its wider application. Both workers as well as organizations strive for efficient instruments when assessing working conditions. Nevertheless, our results indicate that all scales should preferably contain at least three items to allow for a robust assessment of the work environment as scales with fewer items had lower factor loadings, e.g., the scale “organizational restructuring” in the German dataset.

A limitation of our study is the use of cross-sectional data which could have led to an overestimation of the strength of relationships between variables due to common method effects. Particularly, to test the proposition that organizational level factors are antecedents to task level factors requires longitudinal data. Having developed a promising instrument forms the basis for future longitudinal analyses.

Related to the aspect of cross-sectional data is the question of common method variance. The design of our study only allowed for the application of questionnaires and it was not possible to additionally assess data via expert ratings or qualitative interviews. Such additional ways of assessing data would also have allowed us to reduce a possible social desirability bias due to self-reports. We believe the latter was not a major problem in our study because data collection allowed for a high level of anonymity, presumably higher than with probing interviews. Nevertheless, we are convinced that future studies should assess data via different methods in order to allow for a triangulation of results.

Another issue is that organizational level data is best represented by aggregating data at the organizational level. Hence, future research should further test the underlying constructs and their relationships using longitudinal multilevel data.

In addition, further research is needed regarding how the findings on the associations of demand, control, and stability-support at both the task and the organizational level can be translated into actionable public health interventions. While it was not the aim of the current paper to derive such interventions, it gives valuable impulses for designing them.

## 5. Conclusions

In this paper, we showed that the DCS-S-model on the task level could be modeled with three correlated factors across four different countries. Further, at the organizational level—albeit in only one of the four datasets due to a low availability of scales at the organizational level in the other datasets—we confirmed a model with a similar structure. In the one dataset containing sufficient data for both the task and the organizational level, the three organizational level factors for demand, control, and stability-support were antecedents to their three task level analogues. These results lend first support to the concept of trickle-down-effects as proposed by ADC theory.

The JCQ 2.0 scale scores were indicators of the factors in line with theoretical expectations; there were two exceptions to this: skill discretion was an indicator of both task level control and task level demand, and supervisor support was an indicator of organizational rather than task level stability-support. The former result could be shown across all four datasets, rendering it comparably stable. However, it calls for further qualitative investigations in order to find out why this result occurred. The latter result could only be shown in one dataset because the other three datasets did not contain sufficient data to establish such a complex model. Hence, this result calls for further replication and cannot be considered a final result as of yet.

Establishing three factors each on both levels in a first step constitutes a foundation for aggregating scale scores into composites for each factor for future analyses and for testing the predictive and criterion validity of the scales and constructs in studies to come. If the JCQ 2.0’s structure can be replicated in upcoming studies, this will facilitate comprehensive communication of findings in future research. In addition, the multilevel DCS-S framework—now with some empirical support for its fundamentals—points to the need for multilevel interventions to improve work design, starting at the organizational level.

## Figures and Tables

**Table 1:** Scales with their number of items used for structural modeling with the JCQ 2.0.

Construct	Scale (Acronym) ^1^	No. of Items			
		Korea	China	Australia	Germany
task level control	skill discretion (skill dis)	3	3	3	3
	decision authority (dec aut)	3	3	3	3
	conducive development (con dev)	n.a.	n.a.	n.a.	3
task level demand	quantitative demands (quant)	5 ^2^	5	4	5
	emotional demands (emo)	3	2	3	2
task level stability-support	supervisor support (super)	2	3	3	3
	co-worker support (cowork)	2	3	3	3
	collective control (collect)	1	2	n.a.	3
	negative acts (neg ac)	n.a.	n.a.	n.a.	2
organizational level control	organizational decision latitude (org decl)	1	3	3	3
	procedural justice (proced)	3	n.a.	3	3
	conducive communication (concom)	n.a.	n.a.	n.a.	3
organizational level demand	organizational reconstruction (restruc)	n.a.	2	3	1
	organizational disorder (disor)	n.a.	n.a.	n.a.	4
organizational level stability-support	psychosocial safety climate (psc)	n.a.	n.a.	4	4
	rewards (reward)	n.a.	n.a.	2 ^3^	2
	consideration of workers’ interests (consid)	n.a.	n.a.	n.a.	2

^1^ The acronym in brackets is used in Figure 1, Figure A1 and Figure A2 to indicate the scale. ^2,3^ One item formulated in a different way. n.a. not available.

**Table 2:** Model fit indices for confirmatory factor analyses on the task level.

Country	Model	χ^2^ (df, *N*); *p*	*CFI*	*RMSEA*	*SRMR*
Germany	G1	705.85 (24, 2326); <0.01	0.884	0.111	0.062
	G2	327.19 (23, 2326); <0.01	0.948	0.075	0.037
Korea	K1	n.e.	n.e.	n.e.	0.041
	K2	46.85 (10, 7290); <0.01	0.933	0.022	0.030
China	C1	n.e.	n.e.	n.e.	n.e.
	C2	59.89 (10, 2114); <0.01	0.935	0.049	0.034
Australia	A1	285.70 (6, 4214); <0.01	0.933	0.105	0.048
	A2	84.65 (5, 4214); <0.01	0.981	0.061	0.026

n.e. not estimated; Model 1: three correlated factors with each scale only loading on one factor; Model 2: three correlated factors with most scales only loading on one factor with one exception: skill discretion was an indicator of both factors demand and control.

**Table 3:** Factor loadings and factor correlations in the final models (G2, A2, C2, and K2) at the task level for the four data sets.

	Germany—Model G2	Australia—Model A2	China—Model C2	Korea—Model K2
Loadings on factor task demands				
Quantitative demands	0.66	0.68	0.39	0.57
Emotional demands	0.56	0.68	0.64	0.51
Skill discretion	0.49	0.31	0.17	0.43
Loadings on factor task control				
Decision authority	0.68	0.67	0.57	0.47
Skill discretion	0.78	0.80	0.74	0.75
Conducive development	0.87	n.a.	n.a.	n.a.
Loadings on factor task stability-support				
Coworker support	0.75	0.58	0.70	0.54
Supervisor support	0.64	0.78	0.52	0.48
Collective control	0.78	n.a.	0.62	0.62
Negative acts	−0.60	n.a.	n.a.	n.a.
Factor correlations				
Task demands—task control	−0.29	−0.15	0.04 ^+^	−0.53
Task demands—task stability-support	−0.44	−0.31	−0.09 ^+^	−0.05 ^+^
Task control—task stability-support	0.61	0.69	0.56	0.47

n.a. not available. All factor loadings and factor correlations are statistically significant except for those marked with ^+^.

## Data Availability

Due to data privacy regulations, the data from Germany, Korea, and China cannot be distributed. Australian Workplace Barometer Data is available at The Australian Data Archive, Australian National University.

## Appendix A. References to Papers in the Special Issue “The Job Content Questionnaire 2.0: A Tool for Measurement of the Psychosocial Work Environment and Sustainable Work Globally”

**A.1** Karasek, R.; Dollard, M.F.; Östergren, P.-O.; Cho, S.-i.; Houtman, I. The Multi-level Job Content Questionnaire 2.0 (JCQ 2.0) and the Associationalist Demand–Control (ADC) Theory for a Sustainable Global Economy. *Int. J. Environ. Res. Public Health* **submitted** (preprint available at https://doi.org/10.20944/preprints202507.1172.v1).
**A.2** Agbenyikey, W.; Li, J.; Cho, S.-i.; McLinton, S.S.; Dollard, M.F.; Formazin, M.; Choi, B.; Houtman, I.; Karasek, R. International Comparative Reliability and Concurrent Validity Assessment of the Multi-level Job Content Questionnaire (JCQ) 2.0. *Int. J. Environ. Res. Public Health* **accepted**.
**A.3** Formazin, M., Dollard, M.F., Choi, B., Li, J., Agbenyikey, W., Cho, S.-i., Houtman, I.; Karasek, R. International empirical validation and value added of the multilevel Job Content Questionnaire 2.0 (JCQ 2). *Int. J. Environ. Res. Public Health* **2025**, *22(4)*, 492; https://doi.org/10.3390/ijerph22040492.
**A.4** Agbenyikey, W.; Karasek, R.; Formazin, M.; Cho, S.-i.; Choi, B.; Dollard, M.F.; Li, J.; Houtman, I. Associations of external-to-work scales of the Job Content Questionnaire (JCQ) 2.0 with relevant outcome measures. *Int. J. Environ. Res. Public Health* **submitted**.
**A.5** Formazin, M.; Martus, P.; Burr, H.; Pohrt, A.; Choi, B.; Karasek, R. The cross-sectional association of scales from the Job Content Questionnaire 2 (JCQ 2.0) with burnout and affective commitment among German employees. *Int. J. Environ. Res. Public Health* **2025**, *22(3)*, 386; https://doi.org/10.3390/ijerph22030386.
